# Exploring the Effect of Resins of Different Origin on the Structure, Dynamics and Curing Characteristics of SBR Compounds

**DOI:** 10.3390/polym16060834

**Published:** 2024-03-18

**Authors:** Michele Pierigé, Francesca Nardelli, Lucia Calucci, Mattia Cettolin, Luca Giannini, Andrea Causa, Francesca Martini, Marco Geppi

**Affiliations:** 1 Dipartimento di Chimica e Chimica Industriale, Università di Pisa, 56124 Pisa, Italy; michele.pierige@phd.unipi.it (M.P.); marco.geppi@unipi.it (M.G.); 2 Istituto di Chimica dei Composti OrganoMetallici, Consiglio Nazionale delle Ricerche, 56124 Pisa, Italy; lucia.calucci@pi.iccom.cnr.it; 3 Centro per l’Integrazione della Strumentazione Scientifica dell’Università di Pisa (CISUP), 56126 Pisa, Italy; 4 Pirelli Tyre SpA, 20126 Milano, Italy; mattia.cettolin@pirelli.com (M.C.); luca.giannini@pirelli.com (L.G.); andrea.causa@pirelli.com (A.C.)

**Keywords:** time-domain NMR, MAS NMR, nuclear relaxation, field cycling NMR, Rouse dynamics, styrene–butadiene rubber, α-methylstyrene/styrene resin, alkylphenol resin, glycerol ester of maleic rosin, sulfur curing

## Abstract

The replacement of synthetic and petroleum-based ingredients with greener alternatives of natural origin is an imperative issue in rubber technology for the tire industry. In this study, a glycerin-esterified maleated rosin resin, derived from natural resources, is examined as a potential tackifier in styrene–butadiene rubber (SBR) formulations. A comparison is made with two synthetic resins commonly used as tackifiers in tire manufacturing: a petroleum-derived aromatic resin and a phenolic resin. Specifically, this research investigates how these resins affect the structure, dynamics, and curing characteristics of SBR compounds, which are strictly related to the mechanical and technological properties of the final products. Moving die rheometer and equilibrium swelling experiments are employed to analyze vulcanization kinetics and crosslink density, which are differently influenced by the different resins. Information on the polymer–resin compatibility is gained by differential scanning calorimetry and dynamo-mechanical analysis, while solid-state NMR methods offer insights into the structure and dynamics of both cured and uncured SBR compounds at the molecular level. Overall, our analysis shows that the resin of vegetal origin has a comparable impact on the SBR compound to that observed for the synthetic resins and could be further tested for industrial applications.

## 1. Introduction

Tackifying resins are essential additives used to enhance the performance and properties of elastomer-based materials in the tire industry. They serve multiple functions in tire compounds, such as improving adhesion, promoting filler dispersion, and aiding the processability of uncured elastomeric compounds [1,2]. Along with the other ingredients, such as fillers and processing oils, tackifying resins play an important role in the so-called “magic triangle” of tire performance [3,4,5,6,7,8], which refers to the simultaneous optimization of three important tire features: rolling resistance, wet grip, and tread life.

Improving the “magic triangle” is now even more challenging due to the requirements imposed by the green transition. Indeed, in recent years, substituting synthetic and petroleum-based ingredients with renewable and more eco-friendly alternatives has emerged as an imperative concern to reduce the environmental footprint in rubber manufacturing [9,10,11,12,13,14,15,16,17,18,19,20,21,22,23,24,25]. In this frame, resins derived from plant sources have been proposed as multifunctional additives for rubber compounds [21,22,23,24,25], and their influence on macroscopic features, such as curing characteristics, rheological, mechanical, and adhesive properties, has been investigated. On the other hand, little attention has been paid so far to the changes occurring at the “microscopic” level. As a matter of fact, the impact of the new additives on the functional properties of the end products stems from microscopic characteristics concerning the structure, dynamics, and inter-component interactions of the polymer network. An extensive comprehension of the structure–property relationship is therefore required for the rational choice of eco-compatible additives that allow the obtainment of final products with optimized performances. 

From the chemical point of view, tackifying resins are low-molecular-weight hydrocarbon polymers with a high glass transition temperature (*T*_g_) and softening point. The influence of a resin on the curing process, as well as on the viscoelastic and mechanical properties, of rubber compounds is strongly related to the specific chemical and physical properties of the resin itself [26,27,28,29,30]. Vulcanization kinetics and curing characteristics are typically analyzed through moving die rheometer (MDR) and equilibrium swelling experiments. Dynamic mechanical analysis (DMA) measurements, including temperature sweep, frequency sweep, and stress–strain experiments, are employed to investigate the effect of resin addition on the viscoelastic and mechanical behavior of the final products. The effectiveness of resins in enhancing the properties of rubber compounds largely depends on polymer–resin compatibility and miscibility, which can be challenging to predict *a priori*, and represents a crucial issue when considering alternative additives. Typically, the miscibility of polymer and resin in a rubber compound is assessed by comparing the *T*_g_ of the compound with those of the pure components, as measured by differential scanning calorimetry (DSC) [31,32,33] or loss factor (*tan*δ) vs. temperature curves obtained through DMA [27,28,29,30,34,35]. In well-mixed compounds, a single *T*_g_ value should be obtained, higher than that of the pure polymer. Conversely, in cases where the resin is not entirely compatible with the polymer, two distinct *T*_g_ values are measured.

Solid-state nuclear magnetic resonance (SSNMR) has established itself as one of the most powerful techniques for the characterization of polymer structure and dynamics [36,37,38]. In the case of elastomeric materials, ^1^H spin–lattice (*T*_1_, *T*_1ρ_), spin–spin (*T*_2_) relaxation times, and residual ^1^H-^1^H dipolar couplings have proved to be valuable tools to disclose the effects of different formulations and vulcanization conditions on the structural and dynamic properties of the polymer network [39,40,41,42,43,44]. In a recent work by some of the authors, SSNMR was employed to study styrene–butadiene rubber (SBR) compounds of technological interest for the tire industry, before and after the addition of the petroleum-derived tackifying resin Kristalex™ 5140 [44]. ^13^C high-resolution SSNMR spectra and analyses of on-resonance ^1^H free-induction decays (FIDs) were used to obtain information on the molecular structure and on the presence of domains with different mobility in the compound. The degree of dispersion of the resin within the polymer matrix on a nanometer scale was assessed by measuring ^1^H *T*_1_ and *T*_1ρ_ relaxation times [44]. Indeed, it is known that these relaxation times are sensitive to the average dimensions of phase domains because of the averaging effect of spin diffusion [45,46]. ^1^H *T*_1ρ_ measurements and ^1^H *Τ*_1_ field cycling (FC) NMR experiments at variable temperature provided insights into the effect of resin on both local segmental dynamics related to glass transition and motions of SBR polymer chains on longer spatial and time scales.

**Figure 1:**
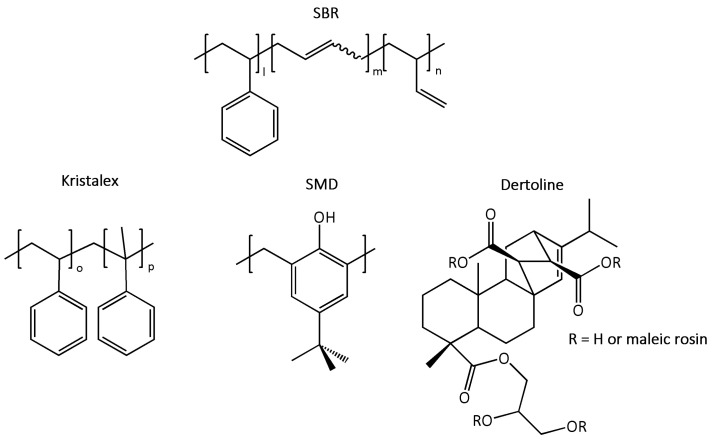
Chemical structures of SBR, Kristalex™ 5140 (Kristalex), SMD-31144 (SMD), and Dertoline MG (Dertoline).

In the present study, a similar SSNMR approach was applied to examine the impact of tackifying resins with different chemical structures on the structural and dynamic features of the polymer network in SBR compounds. Additionally, MDR, equilibrium swelling, DSC, and DMA experiments were conducted to provide insights into vulcanization kinetics, crosslinking degree, polymer–resin compatibility, and tensile properties, and how they are influenced by the type of resin used. In particular, a comparison was drawn between a glycerin-esterified maleated rosin resin of vegetal origin (Dertoline MG) and two synthetic resins commonly used as tackifiers in tire manufacturing, Kristalex™ 5140 and a phenolic resin (SMD-31144). The chemical structures of SBR and of the three resins are shown in Figure 1.

Through the combination of different techniques, the present study provides a comprehensive characterization on how resins with different molecular structure can affect both macroscopic and microscopic properties of SBR compounds. The obtained results can be of valuable help in the design of new rubber formulations of technological interest for the tire industry containing multifunctional and eco-compatible additives.

## 2. Materials and Methods

### 2.1. Materials

All the compounds were provided by Pirelli Tyre SpA (Milano, Italy). Their compositions are reported in Table 1. The polymer matrix consisted of styrene–butadiene rubber (SBR, *M*_n_ = 530,000 g/mol, *M*_w_ = 750,000 g/mol) with a styrene content of 39.5% *w*/*w* and a vinyl content of 38.5% *w*/*w* in the dienic portion, extended with treated distillated aromatic extract (TDAE) as plasticizing oil. Carbon black (CB, N100 series) was added as reinforcing filler. A vulcanization package comprising sulfur, N-cyclohexyl-2-benzothiazole sulfenamide (CBS) as an accelerator, zinc oxide, and stearic acid as an activator was used. Three tackifying resins were employed: Kristalex™ 5140 (Synthomer, Essex, UK, *M*_n_ = 1690 g/mol, *M*_w_ = 4750 g/mol); SMD-31144 (SI Group, Inc., The Woodlands, TX, USA, *M*_n_ = 850 g/mol, *M*_w_ = 1200 g/mol); Dertoline MG (DRT, Dax, France, *M*_n_ = 2000 g/mol, *M*_w_ = 4200 g/mol). Henceforth, we will refer to the employed resins as Kristalex, Dertoline, and SMD, respectively.

The compounds were prepared via a two-step mixing process in a 1.5 L internal mixer. In the first step, SBR was masticated for 30 s; then, the filler was introduced and incorporated within the rubber matrix for 50 s. After that, the resin and all the other ingredients except the vulcanization package were added and further mixing was carried out for 2 min; the dumping temperature of the resulting masterbatch was around 423 K. In the second step, the vulcanization package was added to the masterbatch and mixed for 2 min, reaching a dumping temperature of 373 K to avoid premature crosslinking. Vulcanization was performed at 443 K for 10 min.

The uncured (cured) compounds are denoted as SBR_ref (vSBR_ref), SBR_k (vSBR_k), SBR_d (vSBR_d), and SBR_s (vSBR_s), representing the compound without resin, and those containing Kristalex, Dertoline, and SDM, respectively.

### 2.2. MDR, Equilibrium Swelling, DSC, and DMA Experiments

Curing profiles over time were obtained with a MDR (RPA 2000, Alpha Technologies, London, UK). The experimental conditions were as follows: ±1° oscillation angle, 4.3 bar pressure and 443 ± 1 K for 20 min running time. For each experiment, the following curing properties were measured: minimum torque (*M*_L_ [dNm]), maximum torque (*M*_H_ [dNm]), optimum cure time (*t*_C90_ [s]), and scorch time (*t*_S2_ [s]). The difference between *M*_H_ and *M*_L_ (*M* [dNm]) was then calculated, and the cure rate index (*CRI* [s^−1^]) was determined according to Equation (1) [47]

(1) $$\mathrm{CRI}=\frac{100}{t_{C90}-t_{S2}}$$

The total crosslink density (*M*_c_^−1^, where *M*_c_ is the average molar mass between two adjacent crosslinks) of the vulcanized samples was measured by equilibrium swelling experiments in duplicate. According to the Flory–Rehner method [48], the compounds were weighed three times, once in their pure form, once after being immersed in toluene for 72 h in the dark, and once after being dried overnight in an oven at 343 K under vacuum. *M*_c_^−1^ values were then calculated using the Flory–Rehner equation [48].

Glass transition temperatures (*T*_g_^DSC^) for all compounds and resins were measured by DSC using a Mettler Toledo 823e+ instrument (Mettler-Toledo S.p.A., Milan, Italy). Thermal cycles between 183 and 473 K were performed and the cooling/heating rate was 10 K/min. *T*_g_^DSC^ was assumed as the inflection point of the DSC curve at the heating step.

Temperature sweep tests were performed using an Ares G2 apparatus (TA Instruments, New Castle, DE, USA) by applying a tensile stress mode. Test specimens were prepared by cutting 50 mm × 10 mm rectangular strips from 1 mm thick compound sheets. The temperature dependence of the complex shear modulus was measured by oscillatory shear deformation at a frequency of 1 Hz and at the heating rate of 2 K/min. Then, *tan*δ was calculated as the ratio between the loss modulus (E″) and storage modulus (E′). The glass transition temperature (*T*_g_^DMA^) was assumed as the temperature of the *tan*δ peak.

Tensile tests were carried out using an Instron 5800 apparatus at 298 K and a crosshead speed of 50 mm/min. For each sample, three dumb-bell samples were tested and the average value was evaluated, according to the ISO 37:2017 specifications [49]. Modulus at 10, 20, 50, 100, 200, and 300% of elongation (M10, M20, M50, M100, M200 and M300), tensile strength at break (*TS*_b_), and elongation at break (*E*_b_) were measured, and stored energy density at rupture (SEDR) was calculated as the area under the stress–strain curve.

### 2.3. SSNMR Experiments

^1^H on-resonance FIDs were recorded at a temperature of 303 K using a Niumag permanent magnet working at the ^1^H Larmor frequency of 20.8 MHz interfaced with a Stelar PC-NMR console. The console was equipped with a single-channel static 5 mm probe. ^1^H FIDs were recorded using the mixed magic sandwich echo pulse sequence [50]. The total echo duration was set to 6 (4τ_φ_ + 2τ_90_), where τ_φ_ was 1.5 μs and τ_90_ was 3.3 μs. A total of 200 scans were accumulated, with a recycle delay of 0.5 s for SBR compounds and of 1 s for pure resins. The experimental FIDs were then analyzed by a discrete approach using a non-linear least square fitting procedure implemented in the Mathematica^®^ environment [51].

^1^H spin–lattice relaxation times (*T*_1_) were measured at 20.8 MHz and 303 K applying the inversion recovery pulse sequence coupled with a solid echo pulse scheme. The recovery times ranged from 1 ms to 0.5 s for the cured compounds, and from 1 ms to 1 s for the pure resins. For each experiment, 4–16 scans were acquired, with recycle delays of 0.5 and 1 s for vulcanized samples and pure resins, respectively. 

^1^H FC NMR experiments for the measurement of ^1^H *T*_1_ at variable Larmor frequency in the range of 0.01–35 MHz were carried out using a Spin Master FFC-2000 FC NMR relaxometer (Stelar SRL, Mede, Italy) in the 303–373 K temperature interval. For these measurements, samples were cut into small pieces and loaded into a 10 mm NMR glass tube. The sample temperature was controlled within ±0.1 K using a Stelar VTC90 variable-temperature unit. Above 12 MHz, a non-prepolarized pulse sequence was employed, while below this frequency, a prepolarized pulse sequence was used. The polarizing and detection frequencies were set at 25 and 16.3 MHz, respectively. The switching time was 3 ms, and the 90° pulse duration was 10.9 μs. For each experiment, a single scan was acquired, using 16 values of the variable delay. In all cases, the recovery curves could be fitted using a monoexponential function, with errors on relaxation rate (*R*_1_ = 1/*T*_1_) values below 3%. To ensure data accuracy, *R*_1_ values exceeding 1000 s^−1^ were excluded from the analysis.

^13^C experiments and measurements of ^1^H spin–lattice relaxation times in the rotating frame (*T*_1ρ_) were performed on a Bruker Avance Neo 500 spectrometer working at ^1^H and ^13^C Larmor frequencies of 500.13 MHz and 125.76 MHz, respectively, using a double-resonance 4 mm Cross-Polarization (CP)–Magic Angle Spinning (MAS) probe. The ^1^H 90° pulse duration was 4.3 μs. ^13^C CP/MAS spectra were recorded at a MAS frequency of 5 kHz, using a CP contact time of 0.5 ms, and 1000 transients were accumulated with a recycle delay of 4 s. ^1^H *T*_1ρ_ relaxation times were measured under static conditions in the 303–343 K temperature range, by applying a 90° pulse followed by a spin lock pulse with variable duration in the 0.4–20 ms interval. The spin lock field (ω_1_/2π) was 46 kHz. 

## 3. Results and Discussion

### 3.1. MDR, Equilibrium Swelling, DSC, and DMA Experiments

**Figure 2:**
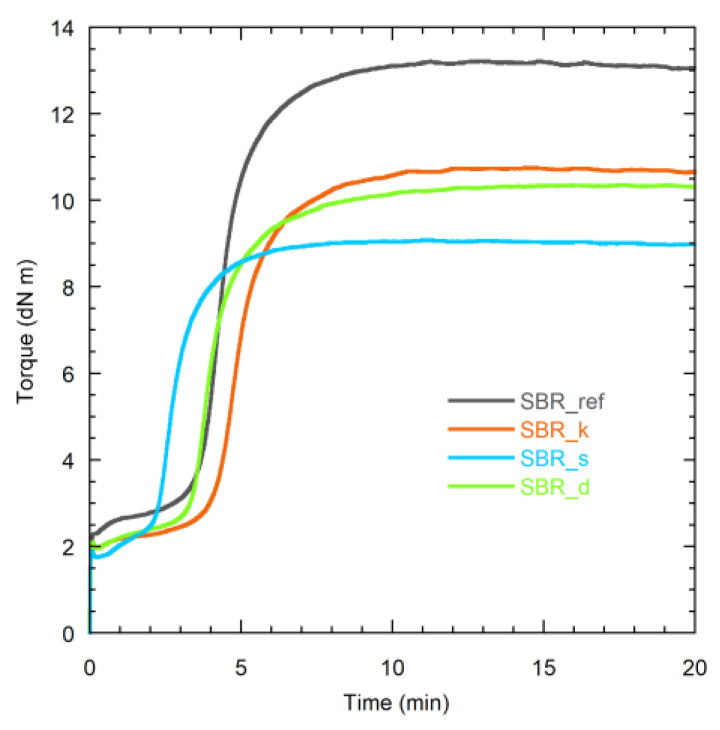
MDR curves of the indicated compounds at 443 K.

Figure 2 shows the MDR curves of the SBR compounds at the vulcanization temperature (443 K), either containing resins or not. The measured curing parameters are summarized in Table 2. Regarding the curing kinetics, it can be noticed that the three resins affect the vulcanization process to a different extent. The beginning of vulcanization is retarded by Kristalex, highly anticipated by SMD, and slightly accelerated by Dertoline, as indicated by the scorch time values (*t*_S2_). A similar trend is observed for the optimal cure time (*t*_C90_). The increase in *t*_S2_ observed in the presence of Kristalex can be attributed to the physical adsorption of curatives onto the resin particles [24,25,35]. Conversely, the reduction in *t*_S2_ induced by SMD and Dertoline is compatible with the fact that these resins contain functional groups capable of accelerating the reaction between accelerators, activators, and sulfur in the early stages of curing [21,25,52]. An alternative explanation could be an improved dispersion of carbon black within the polymer matrix in the presence of these resins. Indeed, it was found that the functional groups present on the surface of carbon black particles could play a catalytic role, promoting the vulcanization reactions [53]. It is worth noticing that the vulcanization rate is strongly increased for SBR_s compared to the other compounds, as highlighted by the *CRI* values (Equation (1)), suggesting that SMD also influences the curing rate.

Regarding the torque values, it can be noticed that the minimum (*M*_L_) and maximum (*M*_H_) torques, as well as their difference (*M*), are lower for the compounds containing the resins. The lower *M*_L_ values obtained for the resin-containing samples indicate that, at the vulcanization temperature, which is higher than the resin’s *T*_g_ (Table 3), all the resins act as plasticizers, effectively decreasing the viscosity of the rubber matrix. This improves the compound’s processability and serves as an initial indication of good compatibility between the resin and SBR [1,24]. On the other hand, the decrease in *M* with the addition of resin suggests a diminished crosslinking efficiency. Indeed, the crosslink density (*M*_c_^−1^) of the cured samples (Table 3) decreases passing from vSBR_ref to the vulcanized compounds containing the resins. The lowest *M*_c_^−1^ values are obtained for vSBR_s and vSBR_d. This finding can be mainly attributed to the deactivation of curatives, particularly accelerators and activators, by adsorption onto the surface of the resin particles. This phenomenon is expected to be more pronounced when the resin contains polar and acidic groups, as in the case of SMD and Dertoline [24,25,35]. 

In Table 3, the *T*_g_ values measured from the DSC curves (*T*_g_^DSC^) of the pure resins and of the uncured and cured compounds are reported. The DSC curves of the SBR compounds are shown in Figure S1. For all the resin-containing samples, a single glass transition temperature is measured, indicating an intimate mixing between SBR and the resin. For the uncured compounds, an increase in *T*_g_ is observed upon resin addition due to the high *T*_g_ values of the pure resins. Resins reduce the available free volume within the SBR matrix, leading to a restriction of polymer chain mobility [35,54]. Upon curing, *T*_g_ increases to a similar extent for all the investigated samples. A rise in *T*_g_ is expected because of the formation of chemical crosslinks [41,43]. However, the decrease in *M*_c_^−1^ with the addition of resin suggests that the observed rise in *T*_g_ is also associated with processes other than crosslinking, occurring during vulcanization in the presence of resin and leading to structural modifications of the polymer chains [43]. Interestingly, these processes appear to be more relevant with SMD and Dertoline compared to Kristalex.

Temperature sweep and stress–strain experiments were carried out on the vulcanized samples to investigate the effect of the different resins on the viscoelastic and tensile properties of the SBR compounds. Temperature sweep experiments were performed in the 193–303 K temperature range, and the obtained *tan*δ curves are shown in Figure S2. The presence of resins induces a shift towards higher temperature of the *tan*δ damping peak associated with glass transition. This result is in line with the restriction of SBR mobility due to the reduced free volume [55]. The values of *T*_g_ determined at the maximum of the *tan*δ peak (*T*_g_^DMA^) show trends with composition similar to those measured by DSC (Table 3).

**Figure 3:**
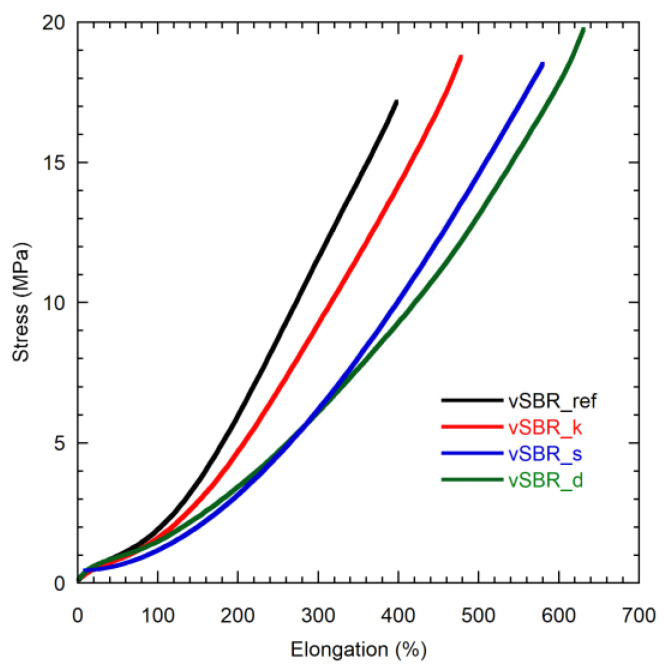
Stress–strain curves of the indicated samples.

Information on the effect of the different kinds of resins on the tensile properties of the vulcanized SBR compounds was obtained from the stress–strain experiments (Figure 3 and Table S1). In all cases, the amount of stress to achieve a certain degree of deformation is lower in the presence of resin, as highlighted by the values of the modulus at 300% elongation (M300). Furthermore, vSBR_d and vSBR_s exhibit lower values of stress for deformation compared to vSBR_k. With the addition of resin, an increase in the values of elongation at break, accompanied by a slight rise in the tensile strength, is also observed. This effect is more pronounced for Dertoline and SMD as opposed to Kristalex. These features can primarily be attributed to the reduction in *M*_c_^−1^ (Table 3) observed when the resin is added, which is higher for vSBR_s and vSBR_d [56,57]. Nevertheless, a plasticization effect of the resins on SBR at high elongations may also play a role [21,25,58]. 

### 3.2. SSNMR Study

#### 3.2.1. Structural Characterization

**Figure 4:**
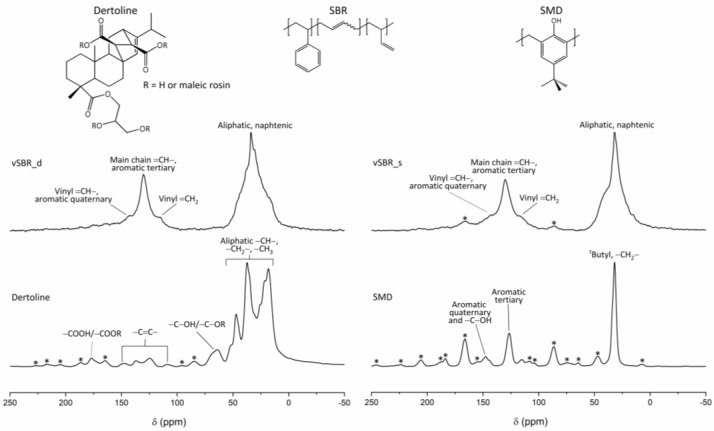
^13^C CP/MAS spectra of Dertoline, SMD, vSBR_d, and vSBR_s with signal assignment. At the top, the chemical structures of SBR and resins are shown. Spinning sidebands are marked with asterisks.

The ^13^C CP/MAS spectra of the pure SMD and Dertoline resins and of the vSBR_s and vSBR_d samples are shown in Figure 4, while those of Kristalex and vSBR_k are reported in a previous publication [44]. In the spectra of pure resins, the observed signals are those expected on the basis of the chemical structure. For instance, in the case of Dertoline, signals typical of an esterified rosin can be observed [59,60,61]. Weak signals ascribable to resin carbons are also visible in the spectra of vSBR_d and vSBR_s, but a detailed analysis is prevented by the superimposition with the much more intense SBR peaks.

**Figure 5:**
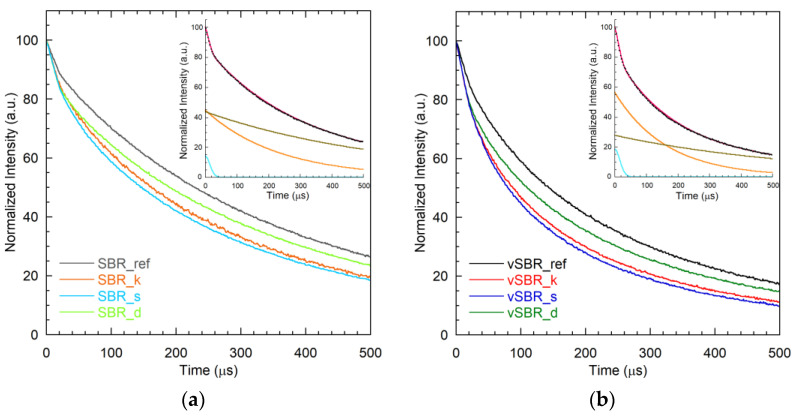
^1^H FIDs of the uncured (**a**) and cured (**b**) SBR compounds. Examples of fitting of the ^1^H FIDs of SBR_d and vSBR_d are shown in the insets, which report the experimental data (black dots), the best-fit function (red), the single Gaussian component (cyan), and exponential *e*1 (orange) and *e*2 (brown) components.

Information on the phase properties of the SBR compounds was obtained by the analysis of ^1^H on-resonance FIDs, reported in Figure 5. The ^1^H FIDs of all the samples were fitted using a linear combination of one Gaussian and two exponential functions following Equation (2):

(2) $$I\left(t\right)=\frac{I\left(0\right)}{100}\left(W_{g}e^{-{\left(\frac{t}{T_{2,g}}\right)}^{2}}+W_{e1\;}e^{-\frac{t}{T_{2,e1}}}+W_{e2\;}e^{-\frac{t}{T_{2,e2}}}\right)$$

where *W_i_* and *T*_2,*i*_ are the weight percentage and the effective spin–spin relaxation time of the *i*-th function, with *i* = *g*, *e*1, or *e*2. Examples of fitting are shown in the insets of Figure 5, while the best-fit parameters are reported in Table 4.

The fast-decaying Gaussian component (*T*_2_,*_g_* of 20–30 μs) can be associated with rigid solid-like domains, while the two long-decaying exponential functions (*T*_2,*e*1_ of 120–300 μs and *T*_2,*e2*_ of 460–770 μs) are ascribable to protons in mobile environments. For the uncured samples, the small fraction of rigid protons detected is assigned to polymer segments involved in either physical entanglements or interactions with the filler particles. A slight increase in the weight of the Gaussian component is observed as a consequence of the addition of resin, which reasonably arises from resin protons. Indeed, the ^1^H FIDs of pure resins are characterized by a short *T*_2_ in the order of 20–40 μs, as shown in Figure S3. This result indicates that the rigid character of the resin is at least partially maintained in the SBR compounds as well. As expected, crosslinking induced a slight increase in the rigid fraction due to the introduction of further topological constraints. The mobile components, accounting for most protons in the samples (82–92%), are ascribable to the polymer chains between topological constraints, as well as to more mobile components from TDAE oil and dangling chains. For both the uncured and cured samples, the presence of resin induced a decrease in both *T*_2,*e*1_ and *T*_2,*e*2_, suggesting a slowdown of polymer chain dynamics. The *T*_2_ values of the exponential components were shorter for the cured samples than for the uncured ones due to the mobility restriction induced by crosslinking.

Information on the degree of mixing between SBR and resins was obtained from measurements of the ^1^H *T*_1_ and *T*_1ρ_ relaxation times. Indeed, spin diffusion tends to average the *T*_1_ and *T*_1ρ_ of protons belonging to domains of the sample with different molecular mobility to one single value if the domain dimensions are lower than 100–200 Å in the case of *T*_1_, and 10–20 Å in the case of *T*_1ρ_. For all samples, one single ^1^H *T*_1_ value was measured at 303 K (Table 5), which was much longer for the pure resins compared to the SBR compounds. The slight increase in *T*_1_ in the resin-containing samples is ascribable to intimate mixing on the 100–200 Å spatial length between SBR and each resin. In the case of *T*_1ρ_, biexponential relaxation curves were obtained for all the resins and SBR compounds, which hamper a straightforward interpretation of the data. The curves were fitted to Equation (3)

(3) $$I\left(t\right)=\frac{I\left(0\right)}{100}\left(W_{a}e^{-\frac{t}{T_{1\rho ,a}}}+W_{b}e^{-\frac{t}{T_{1\rho ,b}}}\right)$$

where *W*_i_ and *T*_1ρ,i_ are the weight percentage and *T*_1ρ_ values of the *i*-th exponential component. The best-fit parameters are reported in Table S2. It is worth noticing that, while for the pure resins the main component of the relaxation curve is characterized by a long *T*_1ρ_ value, a component with such a long *T*_1ρ_ was not detected for the SBR compounds, in agreement with an intimate mixing between SBR and the resin.

#### 3.2.2. Characterization of Dynamics

The characterization of dynamics involved variable-temperature FC NMR measurements of ^1^H spin–lattice relaxation rates (*R*_1_ = 1/*T*_1_) vs. Larmor frequency (ν or ω = 2πν) curves, known as nuclear magnetic relaxation dispersion (NMRD) curves [62,63,64]. In polymers with a high molecular weight at temperatures well above *T*_g_, NMRD curves are primarily influenced by segmental dynamics [65], i.e., local reorientation motions within the Kuhn segments, which are associated with the α-relaxation process connected to the glass transition. At high temperatures and low frequencies, slower and longer-range motions involving larger portions of the polymer chains, referred to as polymer dynamics, also contribute significantly to longitudinal relaxation [65,66], giving rise to characteristic *R*_1_(ω) ∝ ω^−γ^ power law dependences according to the Tube Reptation (TR) model [67,68]. Different values of the γ exponent are expected depending on the regime of polymer dynamics governing relaxation within the observed frequency window. 

**Figure 6:**
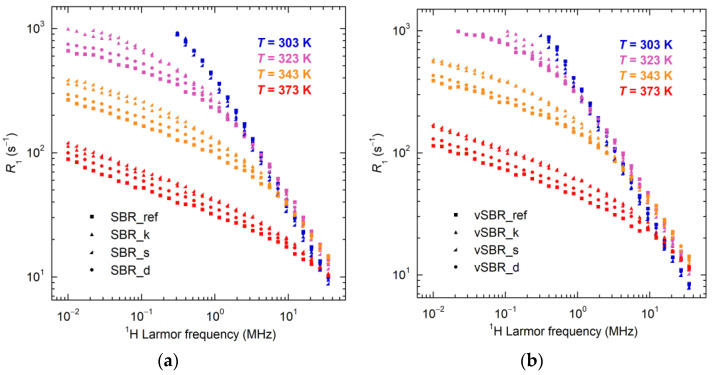
NMRD curves of the uncured (**a**) and cured (**b**) SBR compounds at the indicated temperatures.

Here, NMRD curves were recorded for the uncured and cured SBR compounds in the 0.01–35 MHz Larmor frequency range at different temperatures from 303 to 373 K. A selection of the obtained NMRD curves is shown in Figure 6. For all samples, at *T* < 323 K, *R*_1_ is dominated by segmental dynamics (regime 0 of the TR model). At higher temperatures, two regions with different power law dependences of *R*_1_ on ω can be distinguished: at high frequencies, γ values in the range 0.7–0.8, decreasing by increasing the temperature, are found, due to the overlap of segmental dynamics with the Rouse regime (regime I); at low frequencies, a power law dependence with γ ≃ 0.25–0.28, typical of regime I, is observed. The crossover point between regime 0 and regime I shifts towards lower frequencies as the temperature decreases. Trends of *R*_1_ with decreasing temperature at different frequencies can be explained by the slowdown of segmental dynamics, as described in detail in ref. [41]. At all temperatures, the introduction of resin leads to a shift of the crossover point between the two regimes towards lower frequencies, which follows the order SBR_d (vSBR_d) < SBR_s (vSBR_s) < SBR_k (vSBR_k). Additionally, this shift is accompanied by a decrease in *R*_1_ at high frequencies and an increase at low frequencies. This behavior can be ascribed to the reduction in segmental mobility induced by the presence of resin for both uncured and cured compounds. This effect is less prominent in the case of Dertoline, in agreement with the observed lower increase in *T*_g_ found for SBR_d and vSBR_d. Moreover, a shift in *R*_1_ curves towards a lower frequency is observed on passing from uncured to cured compound due to the slowdown of dynamics induced by crosslinking. 

**Figure 7:**
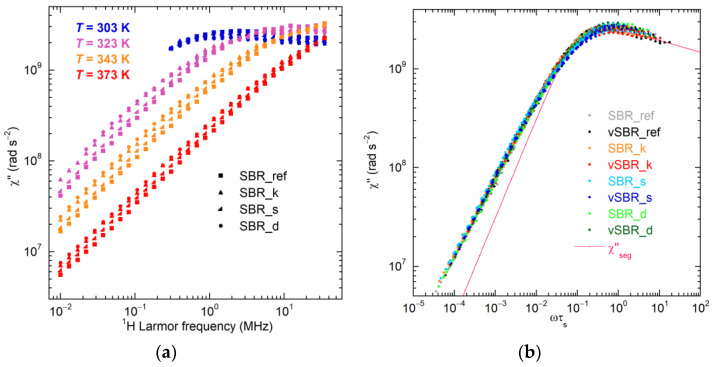
(**a**) χ″(ω) curves at different temperatures of the uncured SBR compounds. (**b**) χ″(ωτ_s_) master curves obtained for the uncured and cured samples. The contribution of sole segmental dynamics (χ″_seg_), calculated on the basis of the Cole–Davidson spectral density function, is shown for comparison.

**Figure 8:**
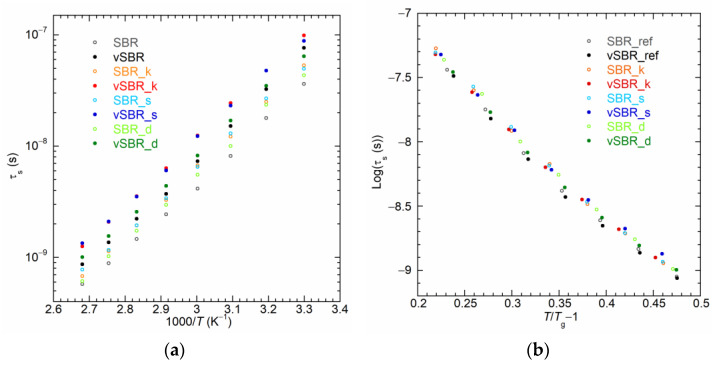
(**a**) Correlation times for segmental dynamics (τ_s_) as a function of inverse of temperature (1000/*T*) and (**b**) Logτ_s_ as a function of (*T*/*T*_g_ − 1) for the indicated samples. *T*_g_^DSC^ values (Table 3) were used in the calculation.

To further investigate the effect of resins on both segmental and polymer dynamics, NMRD curves were converted into NMR susceptibility (χ″(ω) = ω*R*_1_(ω)) representation to construct χ″(ωτ_s_) master curves via the frequency–temperature superposition (FTS) principle [69,70,71,72,73] and, therefore, to determine the correlation times of segmental dynamics (τ_s_), as described in detail in ref. [44]. Figure 7a and Figure S4 show a selection of χ″(ω) curves obtained at different temperatures for the uncured and cured samples, respectively. At the lowest temperatures, the χ″(ω) curves show a maximum corresponding to the condition ωτ_s_ ≃ 1. As the temperature increases, the acceleration of segmental dynamics leads to a shift in the curves towards higher frequencies. Conversely, at each temperature, the addition of resin causes a shift of the χ″(ω) curves towards lower frequencies due to the slowdown of segmental dynamics. In Figure 7b, the χ″(ωτ_s_) master curves are reported together with the contribution of segmental dynamics calculated on the basis of the Cole–Davidson spectral density function [41,74], with the characteristic parameter β_CD_ = 0.12. As shown in Figure 8a, for both the uncured and cured samples, the values of τ_s_ determined from the master curves’ construction increase upon resin addition following the order SBR_ref (vSBR) < SBR_d (vSBR_d) < SBR_s (vSBR_s) < SBR_k (vSBR_k). Moreover, an increase in τ_s_ is observed after curing, attributed to the constriction of segmental mobility caused by the formation of permanent crosslinks. Following a procedure employed in previous works [41,44], the curves of τ_s_ vs. temperature were analyzed in terms of Equation (4), which was obtained by recasting the Vogel–Fulcher–Tammann (VFT) equation [75].

(4) $$\mathrm{Log}\left(\frac{\tau_{s}(T)}{\tau_{0}}\right)=\frac{{\mathrm{Log}}^{2}\left(\frac{\tau_{s}(T_{g})}{\tau_{0}}\right)}{m\left(\frac{T}{T_{g}}-1\right)+\mathrm{Log}\left(\frac{\tau_{s}(T_{g})}{\tau_{0}}\right)}$$

In Equation (4), *m* is the fragility index, τ_0_ is the pre-exponential factor of the VFT equation, and τ_s_(*T*_g_) is the value of τ_s_ at the glass transition, which is set to 100 s [76]. When plotting the values of τ_s_ against the reduced variable $\left(\frac{T}{T_{g}}-1\right)$, it becomes evident that the data from all samples closely follow the same curve (Figure 8b). This result suggests that, as previously found for crosslinking [41], all the examined resins have a negligible impact on the fragility of the polymer at the considered loading and in the investigated temperature range. 

Additional information on dynamics in the kHz frequency regime was obtained by measurements of ^1^H *T*_1ρ_ relaxation times for the cured SBR compounds at a spin lock frequency of 46 kHz as a function of temperature (293–353 K). As mentioned above, all the samples show biexponential recovery curves, which were analyzed using Equation (3). As shown in Table S2, for all the samples, both *T*_1ρ,a_ and *T*_1ρ,b_ increase when increasing the temperature, while *W*_a_ decreases. However, an analysis in terms of individual components might lead to an over-interpretation of *T*_1ρ_ data in terms of dynamics, due to the unclear origin of the biexponential behavior and to the partial averaging operated by spin diffusion. Information on the “averagE″ dynamic behavior of the system under investigation can instead be obtained by considering the population weighted rate average ($R_{1\rho }^{\mathrm{PWRA}}$), calculated as follows:

(5) $$R_{1\rho }^{\mathrm{PWRA}}=\frac{1}{100}\left(\frac{W_{a}}{T_{1\rho ,a}}+\frac{W_{b}}{T_{1\rho ,b}}\right)$$

**Figure 9:**
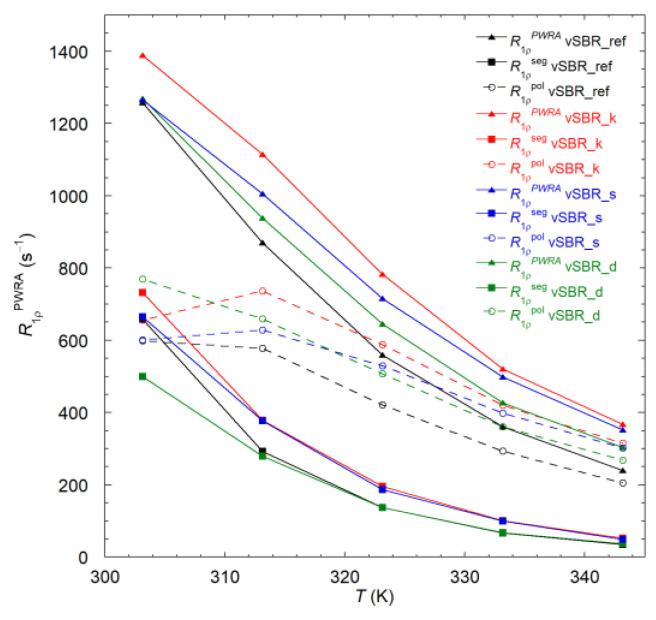
^1^H spin–lattice relaxation rates in the rotating frame, ^1^H $R_{1\rho }^{\mathrm{PWRA}}$ (experimental), $R_{1\rho }^{\mathrm{seg}}$ (calculated), and their difference $R_{1\rho }^{\mathrm{pol}}$ as a function of temperature for the cured SBR compounds (lines are plotted to guide the eye).

As shown in Figure 9, for all the samples, ^1^H $R_{1\rho }^{\mathrm{PWRA}}$ decreases as the temperature rises. An increase in $R_{1\rho }^{\mathrm{PWRA}}$ is observed upon resin addition, following the order vSBR_ref < vSBR_d < vSBR_s < vSBR_k. The observed trends arise from the superimposition of the contributions from segmental and polymer dynamics [44]. Under the hypothesis of statistical independence and time scale separation between segmental and polymer dynamics, and assuming the contribution of the resin is negligible, which accounts for only 10% of the total protons, $R_{1\rho }^{\mathrm{PWRA}}$ can be approximated to the sum of two terms associated with segmental ($R_{1\rho }^{\mathrm{seg}}$) and polymer dynamics ($R_{1\rho }^{\mathrm{pol}}$, according to the following equation:

(6) $$R_{1\rho }^{\mathrm{PWRA}}=R_{1\rho }^{\mathrm{seg}}+R_{1\rho }^{\mathrm{pol}}$$

$R_{1\rho }^{\mathrm{seg}}$ can be calculated according to Equation (7) [36]:

(7) $$R_{1\rho }^{\mathrm{seg}}(\omega ,\omega_{1})=\frac{K_{\mathrm{CD}}}{2}\left[5J_{\mathrm{CD}}\left(\omega \right)+2J_{\mathrm{CD}}\left(2\omega \right)+{3J}_{\mathrm{CD}}\left(2\omega_{1}\right)\right]$$

using the Cole–Davidson spectral density function ($J_{CD}$) with τ*_CD_* = β*_CD_* τ_s_ (β*_CD_* = 0.12) [41] and τ_s_ values obtained from the variable-temperature ^1^H FC NMR experiments (Figure 8). Then, $R_{1\rho }^{\mathrm{pol}}$ can be determined as the difference between $R_{1\rho }^{\mathrm{PWRA}}$ and $R_{1\rho }^{\mathrm{seg}}$. The values of $R_{1\rho }^{\mathrm{seg}}$ and $R_{1\rho }^{\mathrm{pol}}$ calculated at the different temperatures are reported in Figure 9. Interestingly, it can be noticed that, for all the samples, polymer dynamics governs *T*_1ρ_ relaxation at high temperatures. Indeed, at 343 K, $R_{1\rho }^{\mathrm{pol}}$ accounts for about the 87% of the experimental $R_{1\rho }^{\mathrm{PWRA}}$. $R_{1\rho }^{\mathrm{pol}}$ decreases as the temperature is increased, and, except for vSBR_d, a maximum is approached at the lowest temperatures. This trend indicates the presence of Rouse motions with characteristic times in the order of tens of microseconds. The introduction of resin leads to an increase in $R_{1\rho }^{\mathrm{pol}}$ in the order vSBR_ref < vSBR_d < vSBR_s < vSBR_k, indicating a corresponding slowdown of polymer dynamics. Concerning segmental dynamics, for all the samples, $R_{1\rho }^{\mathrm{seg}}$ decreases with increasing temperature. This is consistent with the fact that in the investigated temperature range, ω_1_τ_s_ << 1. Upon resin addition, $R_{1\rho }^{\mathrm{seg}}$ slightly increases in the case of Kristalex and SMD, while no significant variation is observed passing from vSBR_ref to vSBR_d, as expected based on the differences in segmental dynamics observed from *T*_g_ and ^1^H *T*_1_ FC NMR data.

## 4. Conclusions

A glycerin-esterified maleated rosin resin (Dertoline), derived from natural resources, was examined as a potential tackifier in styrene–butadiene rubber (SBR) formulations for the tire industry. Dertoline is compared with two synthetic resins commonly employed as tackifiers, a petroleum-derived aromatic resin (Kristalex) and a phenolic resin (SMD). In particular, the effects of the different types of resins on the structure, dynamics, and curing characteristics of the SBR compounds were investigated by combining rheological, equilibrium swelling, calorimetric, dynamo-mechanical, and solid-state NMR techniques. 

At the investigated loading (15 phr), all the types of resins exhibited good miscibility with SBR on a scale of tens of nanometers. The favorable compatibility between the resin and polymer was further evidenced by the observed plasticization behavior of the resins at the vulcanization temperature, leading to a reduced viscosity and enhanced processability of the compounds.

Regarding the curing characteristics, the addition of resin influenced both the vulcanization kinetics and the degree of crosslinking of the vulcanized samples, to a different extent depending on the type of resin. Similar effects were observed for Dertoline and SMD, which were attributed to the presence of polar groups able to interact with the curing agents. Specifically, these resins seem to contain functional groups able to either promote vulcanization reactions or improve the dispersion of carbon black in SBR, leading to reduced scorch times and, in the case of SMD, to an increased curing rate. On the other hand, the presence of polar groups may cause the adsorption of vulcanizing agents and the occurrence of undesired chemical modifications of the polymer chains during vulcanization, leading to decreased values of crosslink density.

From a microscopic standpoint, each resin retained the rigid character of the pure material when included in the SBR formulations. Conversely, the presence of resin significantly influenced the dynamics of SBR. Specifically, the addition of resin led to a slowdown of both segmental and polymer dynamics in both uncured and cured SBR compounds. This was attributed to the reduction in free volume, which is filled by resin particles. This effect was more pronounced in the cases of Kristalex and SMD compared to Dertoline, in agreement with the *T*_g_ of the pure resins. For the cured SBR compounds, additional contributions arose from chemical crosslinks and structural modifications of the polymer chains, formed during vulcanization, both influenced by the presence of resins. For all the samples, the correlation times of segmental dynamics displayed a Vogel–Fulcher–Tammann dependence on temperature, and no discernible effect of the resins on polymer fragility was observed.

In conclusion, this study offers novel perspectives on how resins with different chemical structures affect both the macroscopic and microscopic properties of SBR compounds for the tire industry. From our analyses, it was possible to show that the resin of vegetal origin has an impact on the SBR compound comparable to that observed with the synthetic resins. This highlights its potential as a promising and environmentally friendly candidate for further testing in industrial applications. The obtained findings can be valuable for the design of new formulations containing multifunctional additives with a reduced environmental footprint.

## Figures and Tables

**Table 1:** Compositions of the investigated SBR compounds in phr (parts per hundred rubber).

Ingredients	SBR_ref	SBR_k	SBR_s	SBR_d
SBR–TDAE	137.5	137.5	137.5	137.5
CB	45	45	45	45
Zinc oxide	3.5	3.5	3.5	3.5
Stearic acid	2	2	2	2
Kristalex	/	15	/	/
SMD	/	/	15	/
Dertoline	/	/	/	15
CBS	4	4	4	4
Sulfur	2	2	2	2
Total	194	209	209	209

**Table 2:** Curing parameters obtained from the MDR curves.

Sample	*M*_H_ (dN m)	*M*_L_ (dN m)	*M* (dN m)	*t*_S2_ (s)	*t*_C90_ (s)	*CRI* (s^−1^)
SBR_ref	13.2	2.3	10.9	226	380	0.65
SBR_k	10.7	1.9	8.8	263	424	0.62
SBR_s	9.1	1.7	7.3	148	269	0.83
SBR_d	10.3	1.9	8.4	216	385	0.59

**Table 3:** *T*_g_ (K) and *M*_c_^−1^ (10^−5^ mol/g) values obtained by DSC (*T*_g_^DSC^) and equilibrium swelling experiments, respectively, for the indicated samples. For the vulcanized samples, the *T*_g_ values measured by DMA (*T*_g_^DMA^) are also reported.

Sample	*T*_g_^DSC^	*T*_g_^DMA^	*M*_c_^−1^
Kristalex	368	-	-
SMD	362	-	-
Dertoline	349	-	-
SBR_ref	246	-	-
SBR_k	249	-	-
SBR_s	249	-	-
SBR_d	247	-	-
vSBR_ref	253	263	1.80
vSBR_k	257	270	1.38
vSBR_s	256	268	0.96
vSBR_d	253	264	0.94

**Table 4:** Weight percentages (*W*_i_) and corresponding *T*_2,*i*_ values obtained as best-fit parameters from the analysis of the ^1^H FIDs at 303 K of both cured and uncured SBR compounds.

Sample	*W_g_* (%)	*W_e_*_1_ (%)	*W_e_*_2_ (%)	*T*_2,*g*_ (μs)	*T*_2,*e*1_ (μs)	*T*_2,*e*2_ (μs)
SBR_ref	8	65	27	22	296	773
SBR_k	13	56	31	25	216	607
SBR_s	15	56	29	29	207	632
SBR_d	14	44	42	21	231	584
vSBR_ref	11	59	30	27	183	615
vSBR_k	19	57	24	26	139	535
vSBR_s	18	55	26	28	121	461
vSBR_d	18	55	27	23	164	601

**Table 5:** ^1^H *T*_1_ (ms) measured at 303 K at the ^1^H Larmor frequency of 20.8 MHz.

Sample	*T*_1_
Kristalex	114
SMD	381
Dertoline	315
vSBR_ref	66
vSBR_k	74
vSBR_s	76
vSBR_d	70

## Data Availability

Data are contained within the article.

## Supplementary Materials

The following supporting information can be downloaded at: https://www.mdpi.com/article/10.3390/polym16060834/s1, Figure S1: DSC curves of the investigated uncured (**a**) and cured (**b**) samples; Figure S2: DMA temperature sweep curves of cured samples; Table S1: Modulus at 10, 20, 50, 100, 200, and 300% of elongation (M10, M20, M50, M100, M200, and M300), tensile strength at break (*TS*_b_), elongation at break (*E*_b_), and stored energy density at rupture (SEDR) of cured samples; Figure S3: ^1^H FIDs of SMD (black) and Dertoline (red) at 303 K; Table S2: Best-fit parameters obtained by a biexponential fitting of the experimental ^1^H *T*_1ρ_ recovery curves of the pure resins and SBR compounds at different temperatures (T, K). For each exponential component, the corresponding weight (*W*_i_, %) and *T*_1ρ_ (*T*_1ρ,I_, ms) values found from the fitting are reported; Figure S4: NMR χ″(ω) curves at different temperatures of the cured SBR compounds.

## References

[1] Gwin L.E., Weaver E.J. (1977). A New Dimension in Rubber Compound Tackifiers. J. Elastom. Plast..

[2] Rodgers B., Waddell W., Mark J.E., Erman B., Eirich F.R. (2005). The Science of Rubber Compounding. Science and Technology of Rubber.

[3] Qin X., Wang J., Han B., Wang B., Mao L., Zhang L. (2018). Novel Design of Eco-Friendly Super Elastomer Materials with Optimized Hard Segments Micro-Structure: Toward Next-Generation High-Performance Tires. Front. Chem..

[4] Weng P., Tang Z., Guo B. (2020). Solving “Magic Triangle” of Tread Rubber Composites with Phosphonium-Modified Petroleum Resin. Polymer.

[5] Heinz M., Grosch K.A. (2007). A Laboratory Method to Comprehensively Evaluate Abrasion, Traction and Rolling Resistance of Tire Tread Compounds. Rubber Chem. Technol..

[6] Vleugels N., Pille-Wolf W., Dierkes W.K., Noordermeer J.W.M. (2015). Understanding the Influence of Oligomeric Resins on Traction and Rolling Resistance of Silica-Reinforced Tire Treads. Rubber Chem. Technol..

[7] Bernal-Ortega P., Gaillard E., van Elburg F., Blume A. (2023). Use of Hydrocarbon Resins as an Alternative to TDAE Oil in Tire Tread Compounds. Polym. Test..

[8] Malinova P., Ilieva N., Metodiev V. (2022). Investigation of Elastomers Ratio Influence in the Composites for Truck Tires Treads Production. J. Chem. Technol. Metall..

[9] Spiegel S. (2018). Sustainable Rubbers and Rubber Additives. J. Appl. Polym. Sci..

[10] Boon Z.H., Teo Y.Y., Ang D.T.-C. (2022). Recent Development of Biodegradable Synthetic Rubbers and Bio-Based Rubbers Using Sustainable Materials from Biological Sources. RSC Adv..

[11] Mekonnen T., Mussone P., Khalil H., Bressler D. (2013). Progress in Bio-Based Plastics and Plasticizing Modifications. J. Mater. Chem. A..

[12] Yuan D., Chen K., Xu C., Chen Z., Chen Y. (2014). Crosslinked Bicontinuous Biobased PLA/NR Blends via Dynamic Vulcanization Using Different Curing Systems. Carbohydr. Polym..

[13] Yuan D., Xu C., Chen Z., Chen Y. (2014). Crosslinked Bicontinuous Biobased Polylactide/Natural Rubber Materials: Super Toughness, “Net-like”-Structure of NR Phase and Excellent Interfacial Adhesion. Polym. Test..

[14] Barrera C.S., Cornish K. (2017). Processing and Mechanical Properties of Natural Rubber/Waste-Derived Nano Filler Composites Compared to Macro and Micro Filler Composites. Ind. Crops Prod..

[15] Barrera C.S., Cornish K. (2015). Novel Mineral and Organic Materials from Agro-Industrial Residues as Fillers for Natural Rubber. J. Polym. Environ..

[16] Barrera C.S., Cornish K. (2016). High Performance Waste-Derived Filler/Carbon Black Reinforced Guayule Natural Rubber Composites. Ind. Crops Prod..

[17] Intharapat P., Kongnoo A., Kateungngan K. (2013). The Potential of Chicken Eggshell Waste as a Bio-Filler Filled Epoxidized Natural Rubber (ENR) Composite and Its Properties. J. Polym. Environ..

[18] Pasquini D., de Morais Teixeira E., da Silva Curvelo A.A., Belgacem M.N., Dufresne A. (2010). Extraction of Cellulose Whiskers from Cassava Bagasse and Their Applications as Reinforcing Agent in Natural Rubber. Ind. Crops Prod..

[19] Awais H., Nawab Y., Amjad A., Anjang A., Md Akil H., Zainol Abidin M.S. (2021). Environmental Benign Natural Fibre Reinforced Thermoplastic Composites: A Review. Compos. C Open Access.

[20] Formela K. Strategies for Compatibilization of Polymer/Waste Tire Rubber Systems Prepared via Melt-Blending. Adv. Ind. Eng. Polym. Res..

[21] Bhattacharyya S.K., Parmar B.S., Mukhopadhyay R., Bandyopadhyay A. (2017). Application of the Resin Derived from the Native Euphorbia Caducifolia Haines as Multifunctional Additive in Filled Natural Rubber Compounds. Rubber Chem. Technol..

[22] Liu Y., Wang S., Li X., Wang Q., Zhang Q., Zhu F., Han J. (2021). Effect of Rosin Resin on the Properties of Natural Rubber/Polyester Layered Fibers. J. Macromol. Sci. B.

[23] Lee S.Y., Gan S.N., Hassan A., Terakawa K., Hattori T., Ichikawa N., Choong D.H. (2011). Reactions between Epoxidized Natural Rubber and Palm Oil-based Alkyds at Ambient Temperature. J. Appl. Polym. Sci..

[24] Ünügül T., Erenkaya M., Karaağaç B. (2022). Use of Liquidambar Orientalis Mills in Natural Rubber Compounds as an Alternative to Synthetic Resins. J. Elastomers Plast..

[25] Bhattacharyya S.K., Parmar B.S., Mukhopadhyay R., Bandyopadhyay A. (2015). Analysis of Autohesion and Physico-Mechanical Properties (Multifunctional Behavior) of the Coagulum from the Latex of Euphorbia Caducifolia Haines Vis-à-Vis Comparison against Synthetic Resins in Natural Rubber Compounds. Rubber Chem. Technol..

[26] Class J.B., Chu S.G. (1985). The Viscoelastic Properties of Rubber–Resin Blends. I. The Effect of Resin Structure. J. Appl. Polym. Sci..

[27] Class J.B., Chu S.G. (1985). The Viscoelastic Properties of Rubber–Resin Blends. II. The Effect of Resin Molecular Weight. J. Appl. Polym. Sci..

[28] Class J.B., Chu S.G. (1985). The Viscoelastic Properties of Rubber–Resin Blends. III. The Effect of Resin Concentration. J. Appl. Polym. Sci..

[29] Liang J., Chang S., Feng N. (2013). Effect of C5 Petroleum Resin Content on Damping Behavior, Morphology, and Mechanical Properties of BIIR/BR Vulcanizates. J. Appl. Polym. Sci..

[30] Yin C., Zhao X., Zhu J., Hu H., Song M., Wu S. (2019). Experimental and Molecular Dynamics Simulation Study on the Damping Mechanism of C5 Petroleum Resin/Chlorinated Butyl Rubber Composites. J. Mater. Sci..

[31] Lindemann N., Finger S., Karimi-Varzaneh H.A., Lacayo-Pineda J. (2022). Rigidity of Plasticizers and Their Miscibility in Silica-Filled Polybutadiene Rubber by Broadband Dielectric Spectroscopy. J. Appl. Polym. Sci..

[32] Dae Han C., Kim J., Man Baek D., Gun Chu S. (1990). Viscoelastic Behavior, Order-disorder Transition, and Phase Equilibria in Mixtures of a Block Copolymer and an Endblock-associating Resin. J. Polym. Sci. B Polym. Phys..

[33] Harper M., Tardiff J., Haakenson D., Joandrea M., Knych M. (2017). Tire Tread Performance Modification Utilizing Polymeric Additives. SAE Int. J. Veh. Dyn. Stab. NVH.

[34] Wolf A., Fernandes J.P.C., Yan C., Dieden R., Poorters L., Weydert M., Verge P. (2021). An Investigation on the Thermally Induced Compatibilization of SBR and α-Methylstyrene/Styrene Resin. Polymers.

[35] Thaijaroen W. (2011). Effect of Tackifiers on Mechanical and Dynamic Properties of Carbon-black-filled NR Vulcanizates. Polym. Eng. Sci..

[36] Müller K., Geppi M. (2021). Solid State NMR: Principles, Methods, and Applications.

[37] Saalwächter K. (2007). Proton Multiple-Quantum NMR for the Study of Chain Dynamics and Structural Constraints in Polymeric Soft Materials. Prog. Nucl. Magn. Reson. Spectrosc..

[38] Saalwächter K. (2012). Microstructure and Molecular Dynamics of Elastomers as Studied by Advanced Low-Resolution Nuclear Magnetic Resonance Methods. Rubber Chem. Technol..

[39] Nardelli F., Martini F., Lee J., Lluvears-Tenorio A., La Nasa J., Duce C., Ormsby B., Geppi M., Bonaduce I. (2021). The Stability of Paintings and the Molecular Structure of the Oil Paint Polymeric Network. Sci. Rep..

[40] Della Latta E., Sabatini F., Micheletti C., Carlotti M., Martini F., Nardelli F., Battisti A., Degano I., Geppi M., Pucci A. (2023). Performant Flexible Luminescent Solar Concentrators of Phenylpolysiloxanes Crosslinked with Perylene Bisimide Fluorophores. Polym. Chem..

[41] Martini F., Carignani E., Nardelli F., Rossi E., Borsacchi S., Cettolin M., Susanna A., Geppi M., Calucci L. (2020). Glassy and Polymer Dynamics of Elastomers by ^1^H Field Cycling NMR Relaxometry: Effects of Cross-Linking. Macromolecules.

[42] Nardelli F., Martini F., Carignani E., Rossi E., Borsacchi S., Cettolin M., Susanna A., Arimondi M., Giannini L., Geppi M. (2021). Glassy and Polymer Dynamics of Elastomers by ^1^H Field Cycling NMR Relaxometry: Effects of Fillers. J. Phys. Chem. B.

[43] Nardelli F., Calucci L., Carignani E., Borsacchi S., Cettolin M., Arimondi M., Giannini L., Geppi M., Martini F. (2022). Influence of Sulfur-Curing Conditions on the Dynamics and Crosslinking of Rubber Networks: A Time-Domain NMR Study. Polymers.

[44] Pierigé M., Nerli F., Nardelli F., Calucci L., Cettolin M., Giannini L., Geppi M., Martini F. (2023). Influence of Resins on the Structure and Dynamics of SBR Compounds: A Solid-State NMR Study. Appl. Sci..

[45] Martini F., Hughes D.J., Badolato Bönisch G., Zwick T., Schäfer C., Geppi M., Alam M.A., Ubbink J. (2020). Antiplasticization and Phase Behavior in Phase-Separated Modified Starch-Sucrose Blends: A Positron Lifetime and Solid-State NMR Study. Carbohydr. Polym..

[46] Martini F., Borsacchi S., Spera S., Carbonera C., Cominetti A., Geppi M. (2013). P3HT/PCBM Photoactive Materials for Solar Cells: Morphology and Dynamics by Means of Solid-State NMR. J. Phys. Chem. C.

[47] Surya I., Sukeksi L., Hayeemasae N. (2018). Studies on Cure Index, Swelling Behaviour, Tensile and Thermooxidative Properties of Natural Rubber Compounds in the Presence of Alkanolamide. IOP Conf. Ser. Mater. Sci. Eng..

[48] Saville B., Watson A.A. (1967). Structural Characterization of Sulfur-Vulcanized Rubber Networks. Rubber Chem. Technol..

[49] (2017). Rubber, Vulcanized or Thermoplastic: Determination of Tensile Stress-Strain Properties.

[50] Maus A., Hertlein C., Saalwächter K. (2006). A Robust Proton NMR Method to Investigate Hard/Soft Ratios, Crystallinity, and Component Mobility in Polymers. Macromol. Chem. Phys..

[51] (2010). Mathematica.

[52] Wu J., Xing W., Huang G., Li H., Tang M., Wu S., Liu Y. (2013). Vulcanization Kinetics of Graphene/Natural Rubber Nanocomposites. Polymer.

[53] Hosseini S.M., Razzaghi-Kashani M. (2018). Catalytic and Networking Effects of Carbon Black on the Kinetics and Conversion of Sulfur Vulcanization in Styrene Butadiene Rubber. Soft Matter.

[54] Kumar K.D., Gupta S., Sharma B.B., Tsou A.H., Bhowmick A.K. (2008). Probing the Viscoelastic Properties of Brominated Isobutylene-*co*-p-Methylstyrene Rubber/Tackifier Blends Using a Rubber Process Analyzer. Polym. Eng. Sci..

[55] Hu X., Zhang R., Wemyss A.M., Du A., Bao X., Geng X., Wan C. (2022). Damping and Electromechanical Behavior of Ionic-Modified Brominated Poly(Isobutylene-Co-Isoprene) Rubber Containing Petroleum Resin C5. Ind. Eng. Chem. Res..

[56] Flory P.J., Rabjohn N., Shaffer M.C. (1949). Dependence of Tensile Strength of Vulcanized Rubber on Degree of Cross-Linking. J. Polym. Sci..

[57] Kawahara S., Nishioka H., Yamano M., Yamamoto Y. (2022). Synthetic Rubber with the Tensile Strength of Natural Rubber. ACS Appl. Polym. Mater..

[58] Lim H., Hoag S.W. (2013). Plasticizer Effects on Physical–Mechanical Properties of Solvent Cast Soluplus^®^ Films. AAPS PharmSciTech.

[59] Tavares M.I.B. (2000). ^13^C NMR Study PP/Rosin Blends. Int. J. Polym. Mater..

[60] De Castro D.O., Bras J., Gandini A., Belgacem N. (2016). Surface Grafting of Cellulose Nanocrystals with Natural Antimicrobial Rosin Mixture Using a Green Process. Carbohydr. Polym..

[61] Anderson K.B., Botto R.E. (1993). The Nature and Fate of Natural Resins in the Geosphere-III. Re-Evaluation of the Structure and Composition of Highgate Copalite and Glessite. Org. Geochem..

[62] Kimmich R., Anoardo E. (2004). Field-Cycling NMR Relaxometry. Prog. Nucl. Magn. Reson. Spectrosc..

[63] Anoardo E., Galli G., Ferrante G. (2001). Fast-Field-Cycling NMR: Applications and Instrumentation. Appl. Magn. Reson..

[64] Kimmich R., Kimmich R. (2018). Chapter 1: Principle, Purpose and Pitfalls of Field-Cycling NMR Relaxometry. Field-Cycling NMR Relaxometry: Instrumentation, Model Theories and Applications.

[65] Kimmich R., Fatkullin N. (2004). Polymer Chain Dynamics and NMR. NMR 3D Analysis Photopolymerization.

[66] Stapf S., Lozovoi A., Kimmich R. (2018). Chapter 13. Field-Cycling Relaxometry of Polymers. Field-Cycling NMR Relaxometry: Instrumentation, Model Theories and Applications.

[67] de Gennes P.G. (1971). Reptation of a Polymer Chain in the Presence of Fixed Obstacles. J. Chem. Phys..

[68] Doi M., Edwards S.F. (1986). The Theory of Polymer Dynamics.

[69] Ding Y., Sokolov A.P. (2006). Breakdown of Time-Temperature Superposition Principle and Universality of Chain Dynamics in Polymers. Macromolecules.

[70] Ferry J.D. (1980). Viscoelasticity Properties of Polymers.

[71] Herrmann A., Kariyo S., Abou Elfadl A., Meier R., Gmeiner J., Novikov V.N., Rössler E.A. (2009). Universal Polymer Dynamics Revealed by Field Cycling ^1^H NMR. Macromolecules.

[72] Hofmann M., Herrmann A., Abou Elfadl A., Kruk D., Wohlfahrt M., Rössler E.A. (2012). Glassy, Rouse, and Entanglement Dynamics as Revealed by Field Cycling ^1^H NMR Relaxometry. Macromolecules.

[73] Kariyo S., Brodin A., Gainaru C., Herrmann A., Hintermeyer J., Schick H., Novikov V.N., Rössler E.A. (2008). From Simple Liquid to Polymer Melt. Glassy and Polymer Dynamics Studied by Fast Field Cycling NMR Relaxometry: Rouse Regime. Macromolecules.

[74] Böttcher C.J.F., Bordewijk P. (1978). Theory of Electric Polarization.

[75] Blochowicz T., Gainaru C., Medick P., Tschirwitz C., Rössler E.A. (2006). The Dynamic Susceptibility in Glass Forming Molecular Liquids: The Search for Universal Relaxation Patterns II. J. Chem. Phys..

[76] Kremer F., Schonhals A., Kremer F., Schonhals A. (2003). Broadband Dielectric Spectroscopy.

