# Supplementary material for: Exploring the Effect of Resins of Different Origin on the Structure, Dynamics and Curing Characteristics of SBR Compounds
Source: Polymers (Basel). 2024 Mar 18;16(6):834. doi: 10.3390/polym16060834 (PMC10975435; doi:10.3390/polym16060834)
Supplement: Supplementary file 1 [file polymers-16-00834-s001.zip › polymers-2876216-supplementary.pdf]

Supplementary Materials

Exploring the Effect of Resins of Different Origin on the Structure, Dynamics and Curing Characteristics of SBR Compounds

Michele Pierigé [1], Francesca Nardelli [2,*], Lucia Calucci [2,3], Mattia Cettolin [4], Luca Giannini [4], Andrea Causa [4], Francesca Martini [1,3,*] and Marco Geppi [1,2,3]

[1] Dipartimento di Chimica e Chimica Industriale, Università di Pisa, 56124 Pisa, Italy; michele.pierige@phd.unipi.it (M.P.); marco.geppi@unipi.it (M.G.)
[2] Istituto di Chimica dei Composti OrganoMetallici, Consiglio Nazionale delle Ricerche, 56124 Pisa, Italy; lucia.calucci@pi.iccom.cnr.it
[3] Centro per l'Integrazione della Strumentazione Scientifica dell'Università di Pisa (CISUP), 56126 Pisa, Italy
[4] Pirelli Tyre SpA, 20126 Milano, Italy; mattia.cettolin@pirelli.com (M.C.); luca.giannini@pirelli.com (L.G.); andrea.causa@pirelli.com (A.C.)
* Correspondence: francesca.nardelli@pi.iccom.cnr.it (F.N.); francesca.martini@unipi.it (F.M.)

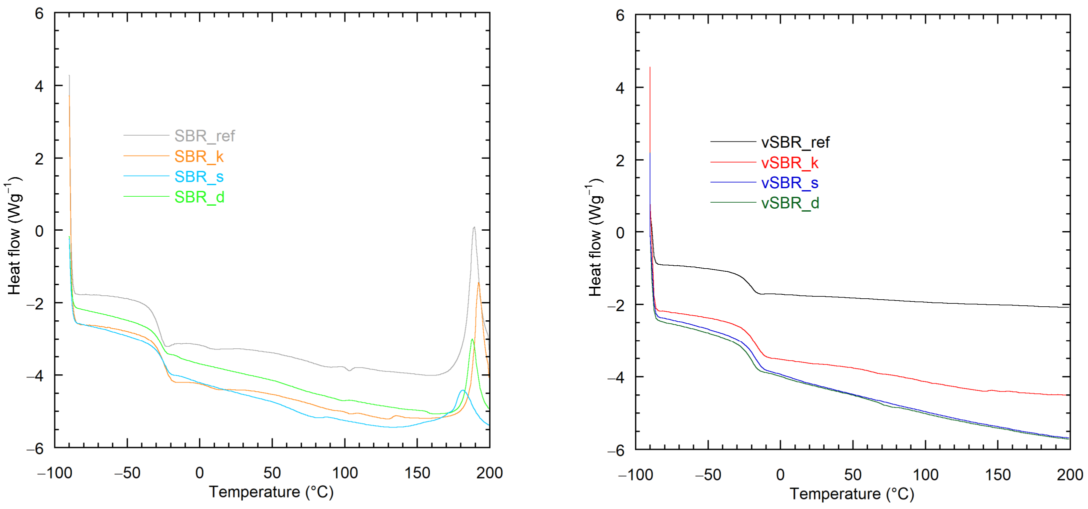

(**a**) (**b**)

Figure S1. DSC curves of the investigated uncured **(a)** and cured **(b)** samples.

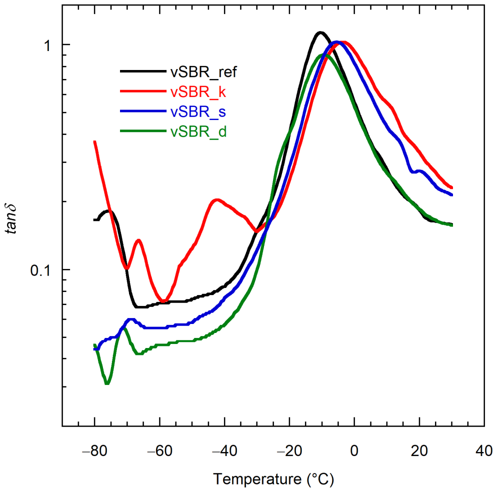

Figure S2. DMA temperature sweep curves of cured samples.

Table S1. Modulus at 10, 20, 50, 100, 200 and 300% of elongation (M10, M20, M50, M100, M200 and M300), tensile strength at break (TS_b), elongation at break (E_b) and stored energy density at rupture (SEDR) of cured samples.

	M10 (MPa)	M20 (MPa)	M50 (MPa)	M100 (MPa)	M200 (MPa)	M300 (MPa)	TS_b (MPa)	E_b (%)	SEDR (MJ/m^3)
vSBR_ref	0.4	0.58	1.01	1.95	5.99	11.53	17	394	26.86
vSBR_k	0.37	0.53	0.9	1.68	4.77	9.29	17.97	467	33.48
vSBR_d	0.57	0.72	1.06	1.64	3.53	6.21	18.6	616	44.35
vSBR_s	0.38	0.51	0.79	1.27	3.18	6.35	18.17	562	38.5

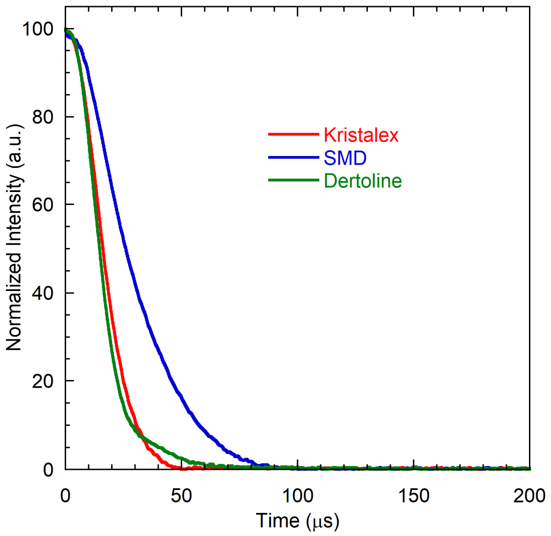

Figure S3. ^{1}H FIDs of the indicated resins at 303 K.

Table S2. Best fit parameters obtained by a biexponential fitting of the experimental [1]H $T_{1\varrho}$ recovery curves of the pure resins and SBR compounds at different temperatures (T, K). For each exponential component, the corresponding weight (W_i, %) and $T_{1\varrho}$ ($T_{1\varrho,i}$, ms) values found from the fitting are reported.

Sample	T	W_a	W_b	$T_{1\varrho,a}$	$T_{1\varrho,b}$
**vSBR_ref**	293	89	11	0.6	4.9
	303	83	17	0.7	4.4
	313	73	27	0.9	5.0
	323	64	36	1.3	6.6
	333	53	47	1.7	8.7
	343	43	57	2.3	11.0
**vSBR_k**	293	90	10	0.6	5.7
	303	87	13	0.6	4.9
	313	79	21	0.7	4.4
	323	70	30	1.0	5.1
	333	60	40	1.3	6.6
	343	50	50	1.6	8.4
**vSBR_d**	293	86	14	0.6	7.5
	303	83	17	0.7	6.5
	313	77	23	0.9	5.9
	323	67	33	1.1	6.3
	333	56	44	1.5	7.6
	343	45	55	1.8	9.4
**vSBR_s**	293	85	15	0.6	4.8
	303	82	18	0.7	4.2
	313	75	25	0.8	4.1
	323	68	32	1.0	4.9
	333	59	41	1.4	6.3
	343	50	50	1.7	8.2
**Dertoline MG**	293	8	92	1.5	16.9
	303	7	93	2.2	18.8
	313	7	93	2.5	20.8
	323	6	94	2.0	22.8
	333	6	94	1.8	24.8
	343	6	94	1.4	25.9
**SMD-31144**	293	3	97	1.0	33.5
	303	4	96	2.1	35.6
	313	4	96	3.0	37.5
	323	5	95	3.3	38.6
	333	4	96	1.5	37.9
	343	4	96	1.8	37.5
**Kristalex™ 5140**	293	8	92	1.4	7.7
	303	8	92	1.3	8.1
	313	7	93	1.3	8.3
	323	8	92	1.2	8.3
	333	8	92	1.3	8.3
	343	9	91	1.3	8.2

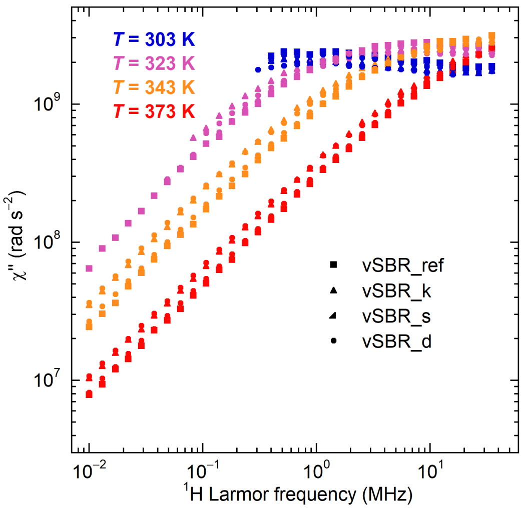

Figure S4. $\chi''(\omega)$ curves at different temperatures of the cured SBR compounds.
